# Associations of Angiotensin-Converting Enzyme Gene Insertion/Deletion (ACE Gene I/D) Polymorphism With Vitiligo: An Updated Systematic Review and Meta-Analysis

**DOI:** 10.7759/cureus.8046

**Published:** 2020-05-10

**Authors:** Mohammad Almohideb

**Affiliations:** 1 Dermatology, College of Medicine King Saud Bin Abdulaziz, University for Health Sciences, Riyadh, SAU

**Keywords:** ace, polymorphis, vitiligo, meta-analysis

## Abstract

Objective

The objective of the article is to summarize the current evidence regarding the association between angiotensin-converting enzyme insertion/deletion (ACE I/D) gene polymorphism and vitiligo disease.

Methods

A computerized search was performed through four electronic databases (PubMed, Scopus, Cochrane Central Register of Controlled Trials [CENTRAL], and Web of Science) with the relevant keywords. Included studies comprised of papers examining the association of ACE gene polymorphisms with vitiligo. Data were pooled as an odds ratio (OR) in random- and fixed-effect models using the Mantel-Haenszel (M-H) method. Review Manager 5.3 software (clicktime.com, Inc., San Francisco, US) was utilized in the meta-analysis.

Results

Ten studies (n=2,740) matching the inclusion criteria were included in the systematic review and meta-analysis. Results showed no significant difference between individuals carrying deletion/deletion (D/D) genotype and individuals with deletion/insertion (D/I) + insertion/insertion (I/I) genotypes in terms of vitiligo risk (odds ratio [OR]=1.13, 95% confidence interval [CI]: 0.78 to 1.64, p=0.53). However, vitiligo risk was higher in the individuals carrying the I/D genotype when compared with individuals with D/D + I/I genotypes (OR=1.29, 95% CI: 1.10 to 1.52, p=0.001). Moreover, the increased risk was observed in individuals carrying D/D when compared with I/I (OR=1.67, 95% CI: 1.33 to 2.09, p<0.0001). D allele was associated with significant risk when compared with the I allele (OR=1.29, 95% CI: 1.15 to 1.45, p<0.0001).

Conclusion

The current evidence suggests that there is a significant association between ACE I/D gene polymorphism and vitiligo. These findings support the use of ACE polymorphism in the prediction of vitiligo as a biomarker.

## Introduction

Vitiligo is a common, acquired, idiopathic, and progressive skin disorder characterized by loss of functional melanocytes and melanin from hair follicles and skin, which leads to one or more white patches of depigmented skin [1, 2]. According to large population surveys, the estimated prevalence of vitiligo was 0.5-2% worldwide, which can reach 8.8% in some areas of Asia, such as in India [3].

Symmetrical and generalized distributions of depigmented skin are the most common presentation of the disease; however, segmental, non-segmental, focal, and mixed distributions are sporadically seen [4]. Despite the idiopathic nature of this disease, autoimmune responses, oxidative stress, and neural factors are the most common factors that are thought to play a role in its pathogenesis [5].

Many researchers identified several vitiligo susceptibility genes that regulate the immune system, such as cytotoxic T-lymphocyte antigen-4 (CTLA4), human leukocyte antigens (HLA), and NACHT leucine-repeat protein 1 [6-8]. One of the important genes is the angiotensin-converting enzyme (ACE) gene, which regulates vascular physiology and participates in the immune system. The D allele of the ACE gene insertion/deletion (I/D) polymorphism was found to be associated with vitiligo [9, 10].

However, the results of the previous studies showed partial disagreement due to multiple factors such as small sample size, racial/ethnic differences, and minor polymorphism effect on vitiligo risk. Therefore, in this systematic review and meta-analysis, the aim was to summarize and investigate the current evidence regarding the association between vitiligo and ACE gene I/D polymorphism.

## Materials and methods

This systematic review and meta-analysis followed the Preferred Reporting Items for Systematic Review and Meta-Analysis (PRISMA) checklist [11].

Search strategy

Computerized searches in four databases (PubMed, Scopus, Web of Science, and Cochrane Central Register of Controlled Trials [CENTRAL]) were done. Research records were exported into EndNote Windows version X8 (Clarivate Analytics, Philadelphia, US). Articles were identified using the relevant search keywords “Vitiligo”, “gene polymorphism”, “Angiotensin-converting enzyme”, “ACE”, and “I/D.”

Selection of studies

Retrieved records were screened in two steps: 1) title and abstract screening, and 2) full-text articles screening for eligibility to meta-analysis. Multiple publications/reports from the same study were excluded, and the latest publication with complete data was considered for meta-analysis.

Inclusion and exclusion criteria

Included studies examined the association of ACE gene polymorphisms with vitiligo. Eligible studies determined the distribution of alleles and⁄or genotypes for the ACE gene I⁄D polymorphism in unrelated cases with vitiligo and in unrelated controls without vitiligo. Studies of other ACE polymorphisms in relation to vitiligo were excluded. Utilized data was from published papers and not from conference abstracts, case reports, or review articles. Moreover, studies were excluded in the following conditions: studies with a population encompassing healthy volunteers, studies with non-reliable data for extraction and analysis, thesis and conference papers, and animal models.

Data extraction

Two independent reviewers extracted the data using an offline data extraction form (Microsoft Excel 2016, windows version). The extracted data include the following: characters of populations and study design (study design, the country, the ethnicity of the study population, the number of cases and controls, demographics of the patients and controls, and the distribution of ACE I⁄D genotypes in the case and control groups).

Data synthesis and heterogeneity

Heterogeneity was assessed and interpreted according to the Cochrane Handbook for Systematic Reviews and Meta-analysis recommendations, by visual inspection of the forest plots and measured by I-square and Chi-square tests [12]. An alpha level (for Chi-square test) below 0.1 indicates significant heterogeneity, and the I-square test is interpreted as follows: 0% to 40% might not be important; 30% to 60% may represent moderate heterogeneity; 50% to 90% may represent substantial heterogeneity. In the case of significant heterogeneity, the random-effects model was utilized. Otherwise, the fixed-effect model was employed. Heterogeneity was resolved using Cochrane’s Leave-one-out method [13].

The Mantel-Haenszel method was chosen above the Peto method due to the relatively few obtained studies with different sample sizes and were not investigating a rare outcome. Studies included subjects from different ethnicities; therefore, a random-effects model was provided in addition to the fixed effects model to examine the change in variability in case of failure to assume that the true effect size was equal across all studies. The odds ratio was pooled in the random effect model meta-analysis (Der-Simonian Liard method). Statistical analyses were performed using Review Manager 5.3 (clicktime.com, Inc., San Francisco, US).

Subgroup analysis

A subgroup analysis was performed according to the following: 1) ethnicity of the included population (Egyptian, Asian, whether Korean or Indian, and European); 2) vitiligo type (generalized, focal, segmental, and non-segmental); and 3) association with other autoimmune diseases.

## Results

Search results

**Figure 1:**
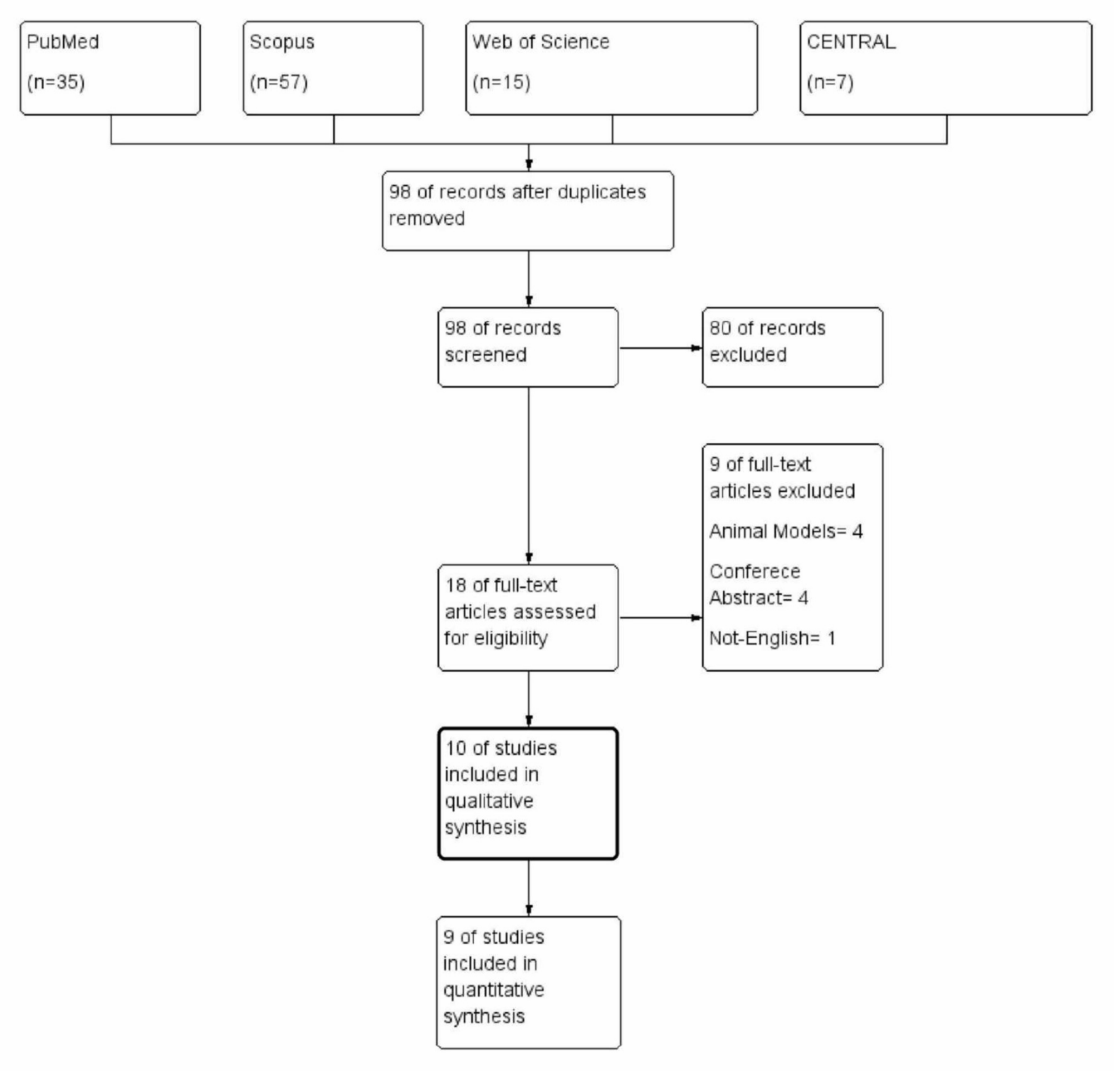
PRISMA flow diagram PRISMA - Preferred Reporting Items for Systematic Review and Meta-Analysis; CENTRAL - Cochrane Central Register of Controlled Trials

Four databases were searched (PubMed, Scopus, Web of Science, and CENTRAL) and records were exported into EndNote. After removing duplicates, 450 unique records are retrieved and ready for screening against the inclusion and exclusion criteria. Title and abstract screening led to exclusion of 412 studies and the finding of 38 potential full-text articles that can be included. After the full-text screening, ten studies (n=2,740) matched the inclusion and exclusion eligibility criteria for this systematic review and meta-analysis [9, 10, 14-22]. The study selection process is shown in the study flow diagram Figure 1.

Demographic characteristics of included studies and individuals

The majority of the included studies are from India (four studies) and Egypt (three studies). The other three studies are from Korea, Turkey, and the UK. The age of the included participants is ranging between two to 85 years. The number of patients with vitiligo was 1,181 and the control group was 1,559. The summary of the included studies is presented in Table 1, and the summary of the demographics and characteristics of included participants is presented in Table 2.

**Table 1:** Summary of included studies PCR - polymerase chain reaction; PCR- RFLP - polymerase chain reaction-restriction fragment length polymorphism; D/D -  deletion/deletion; D/I - deletion/insertion; I/I - insertion/insertion; ACE - angiotensin-converting enzyme

Study	Country	Sample size	PCR	Findings
Jin et al., 2003 [21]	Korea	549	Perkin-Elmer GeneAmp PCR	Significant association of D/D genotype and D allele with vitiligo.
Akhtar et al., 2005 [17]	UK	280	PCR- RFLP	No significant difference in the frequencies of I/I, I/D and D/D genotypes was detected between vitiligo patients and control subjects (p=0.35).
Dwivedi et al., 2008 [20]	India	250	PCR	ACE gene I/D polymorphism may not play a role in the development of generalized vitiligo in Gujarat population.
Deeba et al., 2009 [22]	India	387	PCR	Significant association of D/D genotype and D allele with vitiligo.
Pehlivan et al., 2009 [14]	Turkey	98	PCR–RFLP	No significant differences in either the genotype distribution or allele frequencies of IL4, CCR5 and ACE gene polymorphisms were observed.
Tippisetty et al., 2010 [15]	India	448	PCR	Significant association of I/D genotype with slow progression.
Patwardhan et al., 2013 [9]	India	179	PCR	Serum ACE levels were significantly increased in patients with vitiligo compared with healthy subjects (p<0.0001).
Rashed et al., 2015 [10]	Egypt	149	PCR	ACE gene polymorphism might grant susceptibility to develop vitiligo.
Badran et al., 2015 [18]	Egypt	200	PCR- GeneRuler	ACE gene polymorphism confers susceptibility to vitiligo.
Abdel Azeem et al., 2016 [16]	Egypt	200	PCR- QIAamp DNA mini Kit	The frequencies of both D and I alleles for the ACE genetic marker were significantly different between the control and patient populations (p<0.001).

**Table 2:** Summary of included patients and the genotype distribution SD - standard deviation; D/D -  deletion/deletion; D/I - deletion/insertion; I/I - insertion/insertion

Study	Ethnicity	Case									Control					
		Gender M/F	Age mean ± SD (range)	Total	Clinical type	AD	NAD	D/D	I/D	I/I	Gender M/F	Age mean± SD (range)	Total	D/D	I/D	I/I
Jin et al., 2003 [21]	Asian	51/69	34.5 ± 17.9	120	Generalized/focal (52/68)	9	111	25	66	29	185/244	44.5 ± 16.2	429	59	219	151
Akhtar et al., 2005 [17]	European	44/62	51 (17–85)	106	Generalized (106)	26	80	22	61	23	89/85	43 (19-64)	174	49	88	37
Dwivedi et al., 2008 [20]	Indian	-	-	125	Generalized (125)	0	125	18	61	46	-	-	156	31	74	51
Deeba et al., 2009 [22]	Indian	114/72	30 ± 12.2	186	Segmental/non-segmental (77/109)	0	186	59	83	44	125/76	29 ± 9.8	201	43	84	74
Pehlivan et al., 2009 [14]	European	-	-	48	No classification (50)	-	-	14	25	9	-	-	50	17	23	10
Tippisetty et al., 2010 [15]	Indian	-	-	243	Fast/slow progressive (50/193)	0	243	74	115	54	-	-	205	42	80	83
Patwardhan et al., 2013 [9]	Indian	40/39	29 (2-64)	79	Segmental/non-segmental/focal (6/51/22)	2	77	27	40	12	-	-	100	21	46	33
Rashed et al., 2015 [10]	African	27/47	31.54±15.44	74	Segmental/focal (4/2)	0	74	23	28	23	32/43	34±13.80	75	15	25	35
Badran et al., 2015 [18]	African	40/60	28.46±23.28	100	Segmental/non-segmental/focal (10/64/26)	12	88	32	56	12	32/68	32.16±26.43	100	52	34	14
Abdel Azeem et al., 2016 [16]	African	36/64	33.4±14.7	100	Segmental/focal (2/7)	-	-	62	37	1	35/65	31.1±9.4	100	-	-	-

D/D versus I/I + I/D (recessive model)

**Figure 2:**
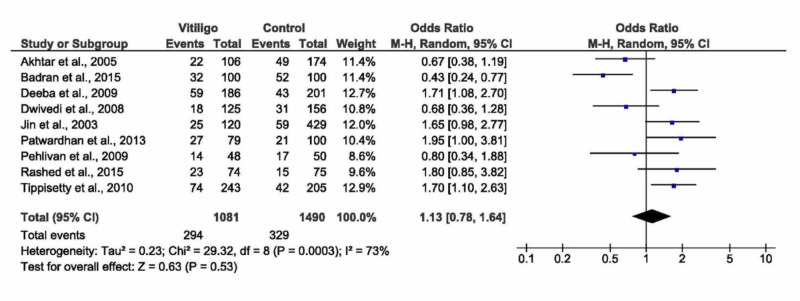
Forest plot of the D/D versus I/I + I/D (recessive model) D/D -  deletion/deletion; I/D - insertion/deletion; I/I - insertion/insertion; CI - confidence interval, M-H - Mantel-Haenszel

The pooled analysis showed that there was no significant association between the deletion/deletion (D/D) versus insertion/insertion (I/I) + insertion/deletion (I/D) (recessive model) and vitiligo in random or fixed effect models (OR=1.13, 95% CI: 0.78 to 1.64, p=0.53; Figure 2, or OR=1.18, 95% CI: 0.98 to 1.42, p=0.09, respectively). The pooled analysis was heterogenous (I^2^=73, p=0.0003). A sensitivity analysis was applied by removing Badran et al. [18], the effect size remained still non-significant (p=0.11); however, the heterogeneity reduced to (I^2^=57, p=0.02), see Table 3. According to the ethnic subgroup, there was a significant association between the recessive model and vitiligo in Asian populations - Indians and Koreans (OR= 1.49, 95% CI: 1.08 to 2.05, p=0.02) with homogenous data (I^2^=45, p=0.12). No significant association was observed in both Egyptians and Europeans (p=0.84 and p=0.15, respectively). In patients with no associated autoimmune disease, a significant association between the recessive model and vitiligo was observed (OR=1.52, 95% CI: 1.04 to 2.24, p=0.03) with homogenous data (I^2^=0, p=0.47). There was no significant difference among all types of vitiligo in terms of association with the recessive model.

D/I versus I/I + D/D

**Figure 3:**
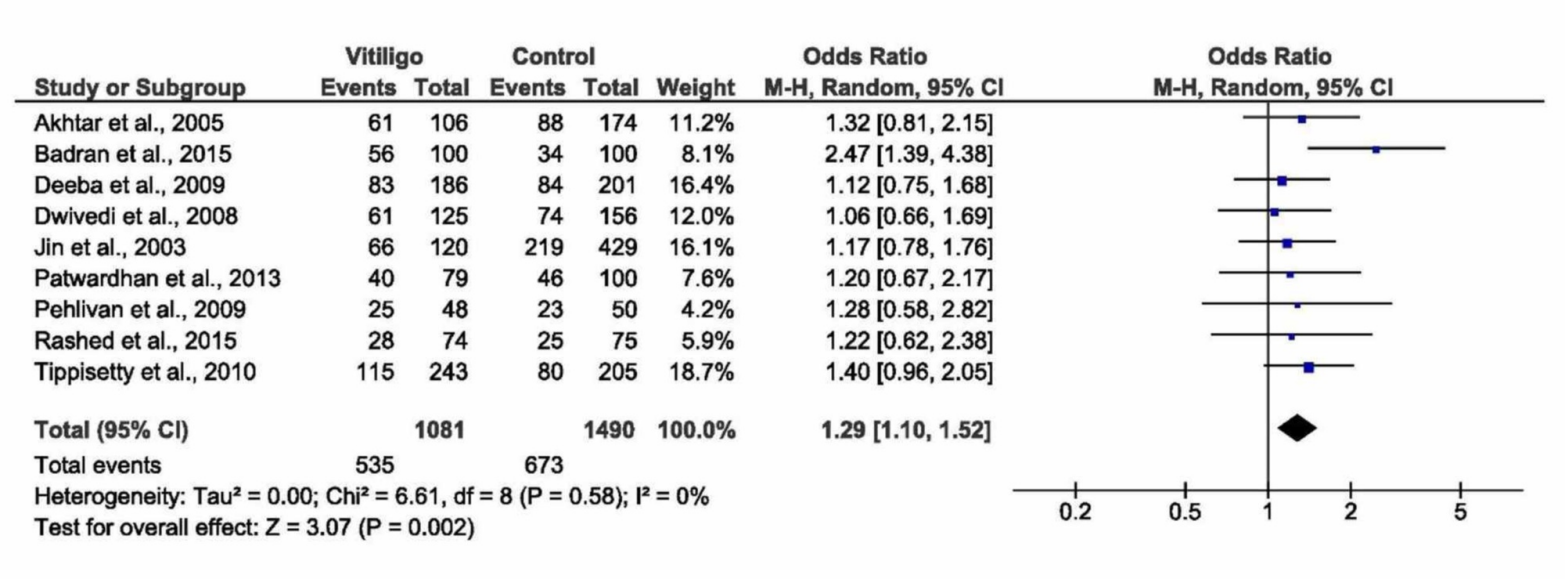
Forest plot of the D/D + I/I versus D/I D/D -  deletion/deletion; D/I - deletion/insertion; I/I - insertion/insertion; CI - confidence interval, M-H - Mantel-Haenszel

Observing D/I versus I/I + D/D, pooled analysis showed a significant association with vitiligo whether by random or fixed effect models (OR= 1.29, 95% CI: 1.10 to 1.52, p=0.002, Figure 3, or OR= 1.29, 95% CI: 1.10 to 1.52, p=0.001, respectively). The pooled analysis was homogenous (I^2^=0, p=0.58), see Table 3. Ethnic subgroups showed a significant association between D/I versus I/I + D/D and vitiligo in the Egyptian population (OR= 1.83, 95% CI: 1.19 to 2.83, p=0.006). No significant association was observed in both Asians and Europeans (p=0.07 and p=0.20, respectively). There was no significant association between D/I versus I/I + D/D and vitiligo in both populations with/without autoimmune disease. Moreover, there was no significant difference among all subtypes of vitiligo in terms of association with D/I versus I/I + D/D; however, the overall effect size showed a significant association with vitiligo as a whole (OR= 1.27, 95% CI: 1.06 to 1.51, p=0.008).

**Table 3:** Summary of a meta-analysis of ACE I/D polymorphism with vitiligo risk D/D -  deletion/deletion; I/D - insertion/deletion; I/I - insertion/insertion; D/I - deletion/insertion; ACE - angiotensin-converting enzyme; OR - odds ratio; CI - confidence interval

Comparison	Test of association			Heterogeneity	
	OR	95 % CI	P-value	P-value	I^2^ (%)
D/D versus I/I + I/D					
Fixed model	1.18	0.98 to 1.42	0.09	<0.01	73
Random model	1.13	0.78 to 1.64	0.53	0.0003	73
Sensitivity analysis (Badran et al., 2015 [18])	1.29	0.94 to 1.77	0.11	0.02	57
I/D + D/D versus I/I					
Fixed model	1.60	1.33 to 1.92	<0.00001	0.03	53
Random model	1.55	1.17 to 2.06	0.002	0.03	53
Sensitivity analysis (Dwivedi et al., 2008 [20])	1.74	1.37 to 2.21	<0.00001	0.23	25
D/I versus D/D + I/I					
Fixed model	1.29	1.10 to 1.52	0.001	0.58	0
Random model	1.29	1.29 to 1.52	0.002	0.58	0
D/D vs I/I					
Fixed model	1.67	1.33 to 2.09	<0.0001	0.001	69
Random model	1.54	1.00 to 2.37	0.05	0.001	69
D versus I allele					
Fixed model	1.29	1.15 to 1.45	<0.0001	<0.0001	77
Random model	1.24	0.97 to 1.59	0.08	<0.0001	77
Sensitivity analysis (Dwivedi et al., 2008 [20] and Badran et al., 2015 [18])	1.45	1.18 to 1.77	0.0003	0.03	56

D ⁄D and I ⁄D versus I ⁄I (dominant model)

**Figure 4:**
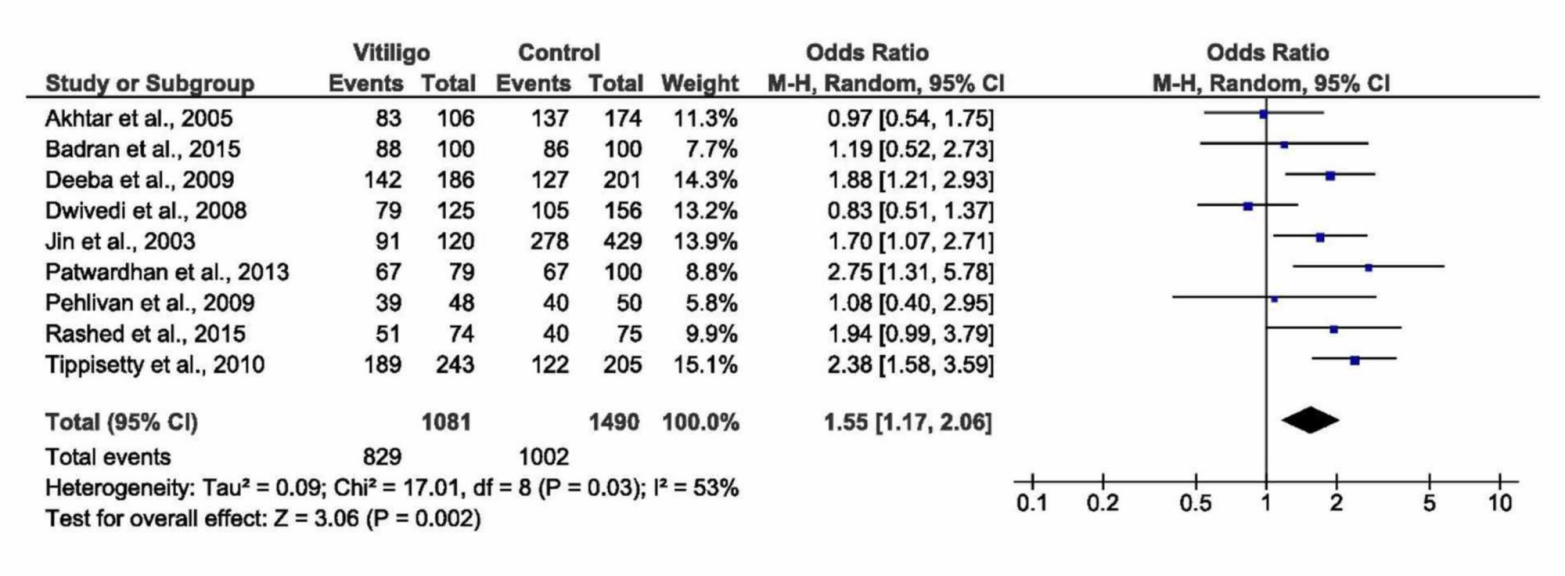
Forest plot of the I/I versus D/D + D/I (dominant model) D/D -  deletion/deletion; D/I - deletion/insertion; I/I - insertion/insertion; CI - confidence interval, M-H - Mantel-Haenszel

Regarding the dominant model, the pooled analysis demonstrated a significant association with vitiligo whether by random or fixed effect models (OR=1.55, 95% CI: 1.17 to 2.06, p=0.002, Figure 4 or OR= 1.60, 95% CI: 1.33 to 1.92), p<0.00001, respectively). The pooled analysis showed moderate heterogeneity (I^2^=53, p=0.03). Heterogeneity was unraveled by performing a sensitivity analysis removing Dwivedi et al., [20] study (I^2^=23, p=0.23), see Table 3. According to the ethnic subgroup analysis, there was a significant association between the dominant model and vitiligo in the Asian populations - Indians and Koreans (OR= 1.74, 95% CI: 1.18 to 2.56, p=0.005), with moderate heterogeneity (I^2^=68, p=0.02). No significant association was observed in both Egyptians and Europeans (p=0.08 and p=1.00, respectively). There was no significant association between the dominant model and vitiligo in both populations with/without autoimmune disease. Moreover, there was no significant difference among all subtypes of vitiligo in terms of association with the dominant model; however, the overall effect size showed a significant association with vitiligo in general (OR= 1.63, 95% CI: 1.10 to 2.41, p=0.02).

Homozygous (D ⁄D versus I ⁄I)

**Figure 5:**
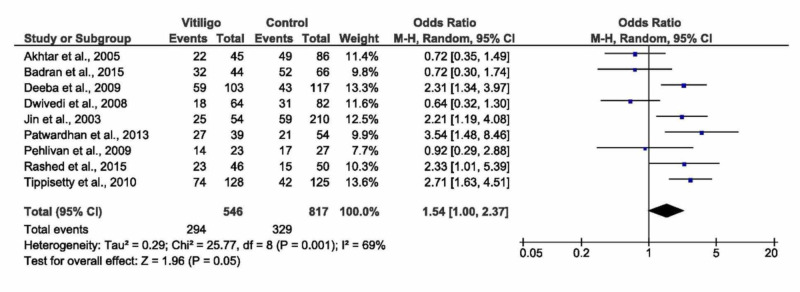
Forest plot of the D/D versus D/I (homozygous model) D/D -  deletion/deletion; D/I - deletion/insertion; CI - confidence interval, M-H - Mantel-Haenszel

The random model of the pooled analysis showed no significant association between homozygous genotypes and vitiligo (OR= 1.54, 95% CI: 1.00 to 2.37, p=0.05, Figure 5). While in the fixed effect model, a highly significant association was observed (OR= 1.67, 95% CI: 1.33 to 2.09, p<0.0001). The pooled analysis demonstrated moderate heterogeneity (I^2^=69, p=0.001), which was not solved by sensitivity analysis, see Table 3. Ethnic subgroup analysis showed a significant association between homozygous genotypes and vitiligo in the Asian populations - Indians and Koreans (OR= 1.99, 95% CI: 1.19 to 3.33, p=0.008). No significant association was observed in both Egyptians and Europeans (p=0.65 and p=0.41, respectively). There was no significant association between homozygous genotypes and vitiligo in both populations with/without autoimmune disease. However, homozygous genotypes were associated with non-segmental vitiligo (OR= 2.35, 95% CI: 1.44 to 3.82, p=0.0006).

D allele versus I allele

**Figure 6:**
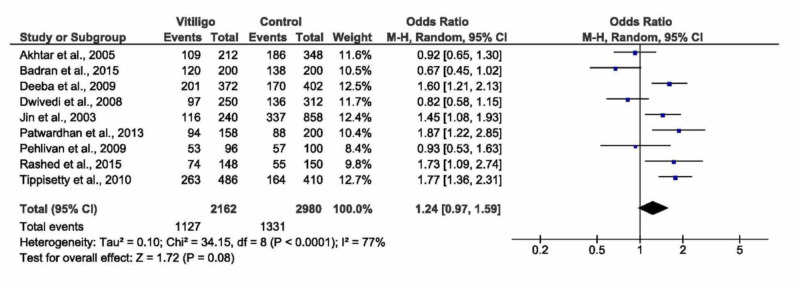
Forest plot of the D allele versus I allele CI - confidence interval, M-H - Mantel-Haenszel

The random-effects model of the pooled analysis showed no significant association between D versus I alleles and vitiligo (OR= 1.24, 95% CI: 0.97 to 1.59, p=0.08), Figure 6. While in the fixed effects model, a highly significant association was observed (OR= 1.29, 95% CI: 1.15 to 1.45, p<0.0001). The pooled analysis demonstrated high heterogeneity (I^2^=77, p<0.0001), which was reduced by sensitivity analysis, see Table 3. Ethnic subgroup analysis demonstrated a significant association between D vs I alleles and vitiligo in the Asian populations - Indians and Koreans (OR=1.44, 95% CI: 1.10 to 1.89, p=0.008). No significant association was observed in both Egyptians and Europeans (p=0.88 and p=0.60, respectively). There was no significant association between D vs I alleles and vitiligo in both populations with/without autoimmune disease. However, homozygous genotypes were once again associated with non-segmental vitiligo (OR= 1.44, 95% CI: 1.07 to 1.95, p=0.02).

## Discussion

The development of vitiligo can be processed by the action of ACE, which participates in degrading the substance P and peptide mediators [23-25]. The presence of substance P can induce many inflammatory reactions like mast cell and leukocyte activation, cytokines production, and plasma extravasation [26]. Moreover, ACE inactivates the bradykinin and the kallikrein-kinin system, which leads to significant implications in the inflammatory process [27].

To the best of our knowledge, this is the largest and most updated systematic review and meta-analysis that evaluated the association between vitiligo and ACE gene polymorphism. Our findings showed that there was no significant difference between the individuals carrying the D/D genotype and the individuals with D/I + I/I genotypes in the term of risk of vitiligo. On the other hand, the risk of vitiligo was higher in the individuals carrying the I/D when compared with individuals carrying D/D + I/I genotypes. Moreover, this risk was also observed in individuals carrying D/D when compared with I/I. D allele was associated with significant risk when compared with the I allele. Remarkably, the I/I genotype has a protective effect against the risk of vitiligo when compared with D/D + D/I genotypes. Asian populations carrying the D/D genotype and D allele have a higher risk of vitiligo when compared with other populations. Egyptians carrying the D/I genotype have a higher risk of vitiligo when compared with other populations. Furthermore, individuals carrying D/D genotype and D allele have a higher risk of non-segmental vitiligo when compared with other types of vitiligo. The observed significant heterogeneity can be explained by the variation between the studies in terms of vitiligo clinical classification, ethnicity, and associated diseases. These findings were supported by published data reporting D allele, and D/D and D/I genotypes associated with a higher risk of vitiligo, especially in Indian populations [9]. In addition, they observed that the serum level of ACE was significantly elevated in vitiligo patients compared with the control group. 

Data from a previous meta-analysis reported a significant association between the recessive model and vitiligo (p<0.0001) [28]. Moreover, they failed to find any association between D/I genotype and vitiligo (p=0.24). This disagreement can be explained by the limited number of studies that they included. Besides, they did not perform a detailed subgroup analysis; they only classified their analysis to overall and generalized vitiligo.

One of the published case-control studies evaluated the distribution of ACE I/D gene polymorphism in the Egyptian population. They reported that the D allele was more distributed in vitiligo patients when compared with control (p<0.001) [14]. In contrast, the I allele was less distributed in vitiligo patients when compared with control (p<0.001). Moreover, they demonstrated that there was no significant difference between patients associated with or without other autoimmune diseases in terms of D/D, D/I, and I/I genotypes (p=0.75) and D and I alleles (p=0.86 and p=0.29, respectively). In contrast, another Egyptian study suggested that individuals carrying the D/I genotype have a higher risk of vitiligo when compared with D/D + I/I [16]. This disagreement between both studies was most likely attributed based on the center of each study.

Despite this study’s significant findings, there are some limitations. This study mainly relied on genetic factors for vitiligo without accounting for environmental factors. In addition, none of the included studies had detailed information about the patients with a family history of vitiligo, which is considered as a major contributor to the occurrence of vitiligo. Moreover, this study showed a significant heterogeneity; however, sensitivity and subgroup analyses were performed to mediate this heterogeneity. 

## Conclusions

In conclusion, the current evidence suggests that there is a significant association between ACE I/D gene polymorphism and vitiligo. This risk may be higher in some populations such as Egyptians and Indias when compared with Europeans. These findings support the use of ACE polymorphism in the prediction of vitiligo as a biomarker.
